# Methylation biomarkers in non-regressive cervical intraepithelial neoplasia grade 2 lesions: an epigenome wide association study

**DOI:** 10.1038/s41416-026-03391-4

**Published:** 2026-04-11

**Authors:** Laura Burney Ellis, Sarah J. Bowden, Maria Paraskevaidi, Deirdre Lyons, Evangelos Paraskevaidis, Anna-Barbara Moscicki, James M. Flanagan, Maria Kyrgiou

**Affiliations:** 1 Department of Metabolism, Digestion and Reproduction, IRDB, Faculty of Medicine, Imperial College London, Du Cane Road, London, W12 0NN UK; 2 Department of Surgery & Cancer, IRDB, Faculty of Medicine, Imperial College London, Du Cane Road, London, W12 0NN UK; 3 Imperial College Healthcare NHS Trust, Hammersmith Hospital, London, Du Cane Road, London, W12 0HS UK; 4 Department of Obstetrics and Gynaecology, University of Ioannina, Ioannina, Greece; 5 Division of Paediatrics, University of California, Los Angeles, USA

**Keywords:** Predictive markers, Cervical cancer, Molecular medicine

## Abstract

**Background:**

DNA methylation has been proposed as a predictive biomarker. Cervical intraepithelial neoplasia grade 2 (CIN2) was historically the cut-off for surgical treatment, however it is increasingly managed with active surveillance, while there is currently no accurate way to predict which lesions will regress.

**Methods:**

We performed the first Illumina 850k array on DNA from serial liquid based-cytology cervical samples from young women with CIN2 that were managed with active surveillance (n = 58). Linear regression identified differentially-methylated sites at baseline distinguishing regressors from non-regressors with persistent or progressive disease at 24-months. Associations with imminent regression and histological change were also evaluated.

**Results:**

We identified three novel differentially-methylated sites; cg12754953 (*ALDH9A1*) methylation was significantly increased at baseline in non-regressors, cg18887759 (*MED25*) methylation was significantly lower in samples from women who regressed within the subsequent 12 months, and cg13556949 (*TULP2*) methylation increased over time between baseline and 12-months of follow-up in non-regressors as compared to regressors.

**Conclusion:**

Methylation could help guide treatment decisions in women considering active surveillance of CIN2 lesions. *ALDH9A1*, *MED25* and *TULP2* may be implicated as genes with possible roles in host response to viral mechanisms. Larger prospective studies are needed to validate these findings.

## Introduction

Although high-risk Human papillomavirus (hrHPV) infection is common, the majority of women will become hrHPV negative within 18 to 24 months [1]. This “clearance” is equated to immune control. The exact immune responses responsible for this have not been fully elucidated but likely include both innate and adaptive arms of the immune system [2]. Persistent infection with oncogenic high-risk Human papillomavirus (hrHPV) can lead to the preinvasive precursor, cervical intra-epithelial neoplasia (CIN) [3, 4]. CIN may lead to cancer in some women if high-grade preinvasive lesions are not detected and treated by cervical screening [5, 6]. Although CIN1 is considered as a state of viral replication [7] commonly managed with active surveillance as most of these lesions regress [8], CIN2 has traditionally been the histological cut-off to proceed to local treatment of the transformation zone of the cervix [8–11].

CIN3 has high rates of progression to invasive cancer of over 50% over 25 years from data reported by one nefarious study of untreated women [12]. CIN2, however, has been labelled as an heterogenous disorder, probably representing the diagnostic capture of both women with worsening and resolving histological changes [13]. A recent meta-analysis reported a rate of regression as high as 60% (95%CI 57-63%) in women younger than 30 [14], and data from Danish population registries found regression rates of 62.9% (95%CI 61.9-63.8%) at 24 months [15]. Given that many of these women are young, active surveillance of CIN2 has been widely adopted in the last decade due to high regression rates and the reproductive risks associated with local excision [16–20]. Although this may still be a preferred option for selected women, concerns have been raised about an increased cumulative risk of invasive cervical cancer. In one study, the risk of invasion was 0.76% [95%CI 0.58-0.95] in those undergoing immediate excision compared to 2.65% [95%CI 2.07-3.23] in those who underwent active surveillance, after 20 years [21]. As such, the decision on surveillance versus immediate treatment needs to balance benefits against the risks of missed invasion and subsequent cancer as well as the need for repeated visits and increased surveillance [22]. In British/European and US guidelines active surveillance is only recommended as the preferred option in women younger than 25 years old [9, 22], and is far more commonly practiced in women younger than 30 [23], although there is no specific age limit to consider its value. At present, there is no available biomarker that can accurately predict which women are likely to regress or not and therefore facilitate individualised treatment of those at risk. Previously proposed biomarkers, such as HPV16 positivity and p16/ki67 immunostaining, have been found to be associated with more severe lesions [24, 25], but not to accurately predict disease progression [26–29]. A predictive molecular biomarker is currently missing from holistic clinical assessment in this setting.

DNA methylation is an epigenetic chemical change involving the addition of a methyl group to specific Cytosine-phosphate-Guanine (CpG) sites [30]. The majority of the physiological function of DNA methylation is to facilitate cellular differentiation by influencing gene expression [31]. DNA methylation can be quantified at specific sites or across the entire genome [32], and it has been noted to become abnormal in cancer [33], with its aberrance proportional to the genetic and histological changes seen in carcinogenesis [34, 35]. Methylation at promoter regions typically results in reduced downstream gene expression [30]; thus methylation at tumour suppressor gene promoters is often observed in cancer [35]. Given that methylation is not only a relatively stable biomarker [36], but functions as a regulatory mechanism, it is biologically plausible that methylation occurs prior to observable histological change [34], and therefore may be ideally suited to the development of a test with real predictive potential [37]. DNA methylation has been shown to be predictive in pre-diagnostic breast cancer samples [38], and is used in glioblastoma prognosis [39].

DNA methylation tests of specific CpG sites or combinations have been proposed as diagnostic markers for high-grade CIN or cervical cancer [40, 41]; DNA methylation is being investigated as a triage marker for hrHPV positive women with promising results [42, 43]. Few studies have assessed methylation tests as predictive markers in CIN2 non-regression. Louvanto et al. assessed methylation of four HPV genes and *EPB41L3* (the S5 classifier) in a cohort of 149 women undergoing conservative management of CIN2 [29], and found moderate accuracy of this marker for prediction of progression, when combined with cytology at baseline. Kremer *at al* found that in a cohort of 75 women with CIN2 or CIN3 who were not treated surgically, those with a negative QIAsure dual-site *FAM19A4/miR124-2* methylation test (Qiagen, Hilden, Germany) regressed more often (74.7%, 95%CI 65.7 – 81.7) than those with a positive methylation test (51.4%, 95%CI 34.6 – 69.5), although this was non-statistically significant [44].

These studies hint at the potential of methylation testing, although a very limited number of CpGs are assessed. Epigenome-wide testing has been made available through array platforms such as Illumina, which provides information on over 850,000 CpG sites for exploratory epigenome-wide association study (EWAS). EWAS has identified novel predictive methylation biomarkers associated with cancer development, progression, or prediction of treatment response in other cancers, including in breast [38], prostate [45] and ovarian [46]. We have recently identified novel diagnostic CpG sites in CIN3 and cervical cancer using EWAS [47]. Another EWAS identified 336 further novel differentially methylated sites that may predict progression of normal or CIN1 to CIN2 or worse [48].

In this study, we conducted the first EWAS of histologically-confirmed CIN2 lesions that were managed with active surveillance and explored novel CpG sites associated with non-regression, imminent regression, and sites that changed significantly over time in line with histological change.

## Materials and methods

### Study participants

Women aged 13-24 with histologically-confirmed CIN2 were recruited from 12 participating clinics in Kaiser Permanente, Northern California (KPNC), United States of America, (study approved by the institutional review board of the University of California, San Francisco (UCSF) and KPNC). Women with immunosuppression or pregnancy were excluded, or those planning to leave the area in the subsequent 3 years. The included cohort was one of the first to undergo a trial of active surveillance with serial sampling every 3-6 months for up to 3 years [28]. Clinical features such as size of the lesion were not recorded, but all women were deemed clinically suitable for a trial of active surveillance. Informed consent was obtained from all subjects. Histological samples were reviewed twice, at both KPNC and UCSF, by a single pathologist at each site for consistency. Any sample progressing to CIN3 was confirmed by a third pathologist. We analysed a subset of this group including 58 women where there was sufficient DNA for analysis. Regression was defined by normal histology, or by at least two consecutive cytologically normal samples at least 3 months apart, where histology was not available. Non-regression was defined as persistent CIN2 at 24 months or progression to CIN3. For each woman, we selected paired samples: one at the time of CIN2 diagnosis (baseline) and one after approximately 12 months (follow-up), to make a total of 116 samples.

### Sample processing and methylation analysis

For each sample, 1 ml of liquid-based cytology (LBC) was taken at either baseline or follow-up, and stored at -80 degrees. LBC samples underwent DNA extraction with an LBC-adapted protocol. DNA was quantified using Qubit (Thermofischer Scientific). DNA samples were diluted with DNase-free water or concentrated using SpeedVac (Savant) to achieve 200 ng DNA in 40ul DNase-free water.

After bisulphite conversion, an Illumina Methylation EPIC BeadChip 850k Methylation array version 1 ( > 900,000 CpG sites) was performed on 116 samples. Samples were evenly distributed across plates and chips by outcome status, and subsequently randomised within plate and chip.

### Data and statistical analysis

No samples failed quality control. Data were pre-processed and normalised using the *minfi* package in statistical software R. The methylation level at each CpG was expressed as *β* values, which represent the percentage of cytosines methylated at that location, and M values, which were obtained from logit_2_-transformation of the *β* value. *β* values and M values were analysed using a bespoke statistical pipeline. Probes that failed in >20% of samples were excluded from the analysis. Methylation of known Single Nucleotide Polymorphisms (SNPs) confirmed the identity of the follow-up sample (Figure S3). Principle Components Analysis (PCA) was used to distinguish confounders to adjust for; plate, position on plate, chip, position on chip, timepoint, status, age, ethnicity, HPV16/18 status, previous pregnancy, smoking status, and previous *Chlamydia trachomatis* or *Neisseria gonorrhoeae* infection were examined.

Logistic regression was conducted to identify any CpG sites associated with firstly, progressors or persistent (“non-regressors”) as compared to all regressors (within 24 months), with the aim of identifying a biomarker at baseline associated with non-regression at 24 months, and therefore clinically, which patients should undergo immediate surgical treatment. Secondly, we aimed to identify a marker of imminent regression, i.e we compared samples which progressed or were stable (non-regressors) as compared to the samples that regressed within the following 12 months (immediate regressors’ baseline sample, and late regressors’ 12-month follow-up sample). Thirdly, the Δ, or delta (change in methylation between the baseline and follow-up samples) was calculated and after adjusting for confounders (as isolated in a delta-specific PCA), logistic regression was employed to identify CpG sites within the delta associated with progressors as compared to all regressors. M values were used for logistic regression in the analyses for progressors versus regressors, and regression within 12 months, and *β* values were used for the delta analysis, as these were the most normally distributed.

False Discovery Rate (FDR) was used to correct for multiple testing. Due to a small sample size, we also applied a common threshold (p < 0.00001) to both *β* values and M values in the two main analyses, to identify any novel CpG sites that were identified in the linear regression but failed to meet FDR significance.

## Results

The included cohort had a high rate of regression. Of the 58 women, 12 of 58 (21%) patients were classed as non-regressors at 24 months, which consisted of patients with both persistent (6/58, 10%) and progressive lesions (6/58, 10%). 46 of 58 (79%) women were classed as regressors at 24 months. Of the regressors, 31/46 (67.4%) regressed within 12 months (immediate regressors) and 15/46 (32.6%) regressed between 12 and 24 months (late regressors).

Patient characteristics are described in Table 1. All 58 women had a history of previous sexual intercourse, and were positive for hrHPV at baseline. The patients, when divided into non-regressors or regressors (total, or immediate and late) were not statistically significantly different in distribution of age, ethnicity, HPV16/18 status, previous pregnancies, smoking status, and previous history of *Chlamydia trachomatis* or *Neisseria gonorrhoeae* infection.

**Table 1:** Included participant characteristics

		Total N = 58	Non-regressors N = 12 n/N (%)	All regressors N = 46 n/N (%)	_p_1	Regressors; immediate and late		_p_2
						Immediate regressors (N = 31) n/N (%)	Late regressors (N = 15) n/N (%)	
Mean age in years (range)		20.7 (16.1-25.0)	20.7 (16.3-24.8)	20.6 (16.1-25.0)	0.9771	20.6 (16.1-25.0)	20.7 (17.6-24.5)	0.8681
Race	Asian/Pacific Islander	3	1/12 (8)	2/46 (4)	0.808	1/31 (3)	1/15 (7)	0.9481
	Black or African American	9	2/12 (17)	7/46 (15)		4/31 (13)	3/15 (20)	
	Hispanic /Latino	12	2/12 (17)	10/46 (22)		6/31 (19)	4/15 (27)	
	Mixed	8	3/12 (25)	5/46 (11)		3/31 (10)	2/15 (13)	
	White	21	4/12 (33)	17/46 (37)		12/31 (39)	5/15 (33)	
	Missing data	5	0/12 (0)	5/46 (11)		5/31 (16)	0/15 (0)	
Human Papillomavirus (HPV) status	HPV 16/18 positive	26	7/12 (58)	19/46 (41)	0.4971	15/31 (48)	4/15 (27)	0.1892
	HPV 16/18 negative	26	4/12 (33)	22/46 (49)		12/31 (39)	10/15 (67)	
	Missing data	6	1/12 (8)	5/46 (11)		4/31 (13)	1/15 (7)	
Parity	Previous pregnancy	25	8/12 (67)	17/46 (37)	0.2265	12/31 (39)	5/15 (33)	0.6358
	No previous pregnancy	28	4/12 (33)	24/46 (52)		14/31 (45)	10/15 (67)	
	Missing data	5	0/12 (0)	5/46 (11)		5/31 (16)	0/15 (0)	
Smoking status	Smoker	14	3/12 (25)	11/46 (24)	1	9/31 (29)	2/15 (13)	0.2709
	Non-smoker	26	6/12 (50)	20/46 (43)		11/31 (35)	9/15 (60)	
	Missing data	18	3/12 (25)	15/46 (33)		11/31 (35)	4/15 (27)	
STI status	History of previous STI	16	5/12 (42)	11/46 (24)	0.5305	10/31 (32)	1/15 (7)	0.0647
	No history of previous STI	37	7/12 (58)	30/46 (65)		16/31 (52)	14/15 (93)	
	Missing data	5	0/12 (0)	5/46 (11)		5/31 (16)	0/15 (0)	

Non-regressors included those managed with active surveillance of Cervical Intraepithelial Neoplasia grade 2 (CIN2) that did not regress; i.e. persistent or progressive disease. Immediate regressors regressed within the first 12 months of active surveillance, and late regressors after 12 but before 24 months.

*HPV* Human papillomavirus, *STI* sexually-transmitted infection (Chlamydia trachomatis or Neisseria gonorrhoeae).

*p*-value calculated using student’s t test. 1 =student’s t test comparing characteristics for non-regressors and regressors. 2 = student’s t test comparing characteristics for immediate regressors and late regressors.

All 116 samples met quality control. There was no significant difference in the overall distribution of *β* values between samples, including when stratified by status (Supplementary Figures – Fig. S1), or by timepoint (Fig. S2). No samples failed with more than 5% of probes. A SNP heatmap confirmed the identities of both baseline and follow-up samples (Fig. S3). PCA 3, 4, 5, and 8 accounted for variation between plate and chip and were subsequently adjusted for, in addition to previous pregnancy and smoking status (Figs. S4 and S5). QQ plots were calculated for each analysis (Figs. S6-8). The lambda statistic for regression within 24 months was 0.9095 for *β* values, and 0.8892 for M values. For regression within 12 months, lambda was 0.8026 for *β* values and 0.8001 for M values. The full list of statistically significant CpGs associated with outcome for each analysis are displayed in Table 2.

**Table 2:** Differentially-methylated Cytosine-phosphate-Guanine sites identified using Linear Regression of clinical regression status

CpG	Chromosome, position	Relation	Gene	P Value	FDR	Significant in >1 analysis
Regression within 24 months (non-regressors vs all regressors)						
cg16024950	7, 100254149	Island	*ACTL6B*	9.12e-06	0.999	
cg12043473	7, 66750978	N Shore	Intergenic	6.95e-06	0.999	
cg08685955	19, 48871461	S Shelf	*SYNGR4*	1.63e-06	0.467	
cg02099267	4, 963838	Island	*DGKQ*	6.80e-06	0.999	*
cg12754953	1, 165656459	Open Sea	*ALDH9A1*	7.90e-08	**0.068**	
Imminent regression (non-regressors vs regressors within subsequent 12 months)						
cg01319583	16, 224928	N Shore	Intergenic	8.77e-06	0.685	
cg15546285	22, 31999222	Open Sea	*SFI1*	6.29e-07	0.270	
cg03402351	16, 11759492	N Shelf	Intergenic	1.28e-06	0.276	
cg11806672	13, 79176608	Island	*POU4F1*	8.33e-06	0.685	
cg02099267	4, 963838	Island	*DGKQ*	9.67e-07	0.276	*
cg03340044	20, 36875438	Open Sea	*KIAA1755*	6.21e-06	0.685	
cg01287871	8, 27525102	Open Sea	*SCARA3*	1.93e-06	0.333	
cg18887759	19, 50324282	S shelf	*MED25*	4.59e-08	**0.040**	
Delta (change in methylation over time: non-regressors vs all regressors)						
cg06616198	5, 994564	Open Sea	LOC100506688	6.47e-06	0.833	
cg13556949	19, 49395260	Open Sea	*TULP2*	3.48e-08	**0.030**	
cg23054188	5, 115886970	Open Sea	*SEMA6A*	2.07e-06	0.833	

*CpG* Cytosine-phosphate-Guanine site, *FDR* False Discovery Rate.

*P*-values were taken from the most normally-distributed results; M values were used for regression within 24 months and imminent regression. β

values were used in the delta analysis. Position = base pairs.

### Regression within 24 months

**Fig. 1:**
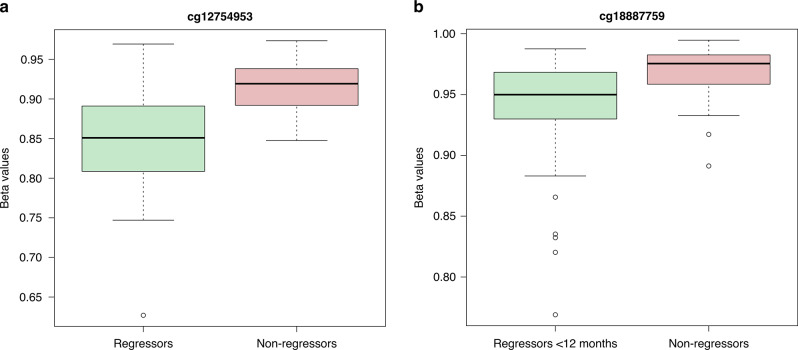
Boxplots showing the level of methylation, in beta values. **a** cg12754953 Regressors vs non-regressors (24 months). **b** cg18887759 Imminent regressors (subsequent 12 months) vs non-regressors.

**Fig. 2:**
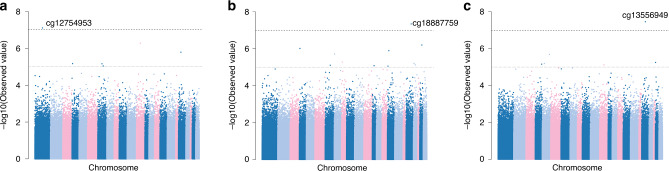
Manhattan plots showing the association between methylation at cytosine-guanine (Cg) sites using generalized linear regression. Cg sites are ordered by their position on the chromosome (x-axis). The *p*-values show the strength of the association are represented on a log-scale (y-axis). Dashed line = False Discovery Rate 0.1. Dotted line = *p* < 10^-5^. **a** M values: Regressors vs Non-regressors (<24 months). **b** M values: Imminent regressors (subsequent 12 months) vs Non-regressors. **c** Beta values: Delta analysis (change in methylation between baseline and follow-up (12 months) sample, Regressors vs Non-regressors.

Using generalised linear regression, for M values in baseline samples in non-regressors as compared to all regressors (within 24 months), there was one CpG site that met statistical significance at an FDR <0.1; cg1275493; *ALDH9A1*; FDR 0.068 (Figs. 1A and 2A). There was no overlap in the interquartile range of beta values between regressors and non-regressors for cg1275493. When a common threshold (p < 0.00001) was applied across *β* values and M values, there were five CpGs that were differentially methylated in non-regressors as opposed to regressors (cg16024950, cg12043473, cg08685955, cg02099267, cg12754953) (Fig. 2A).

### Imminent regression

In an analysis of non-regressing samples as compared to samples regressing imminently (within the following 12 months), there was one CpG site that met statistical significance at an FDR < 0.1; cg18887759; *MED25*; FDR 0.039 (Figs. 1B and 2B). Seven CpGs were differentially methylated in samples that were imminently regressing (cg01319583, cg 15546285, cg03402351, cg11806672, cg02099267, cg03340044, cg01287871) (Fig. 2B) when a common threshold (p < 0.00001) was applied across *β* values and M values.

### Delta (Δ)

**Fig. 3:**
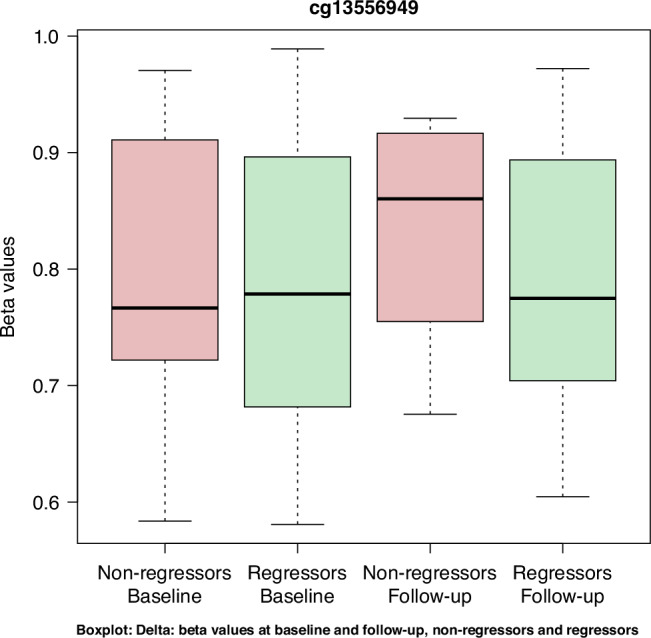
Boxplot showing the level of methylation in beta values for cg13556949, at baseline and follow-up, for non-regressors and regressors.

PCA adjusted for in the delta included position on plate, plate, chip position, chip, ethnicity, time, and HPV16/18 status. In the delta analysis, one CpG site was differentially methylated at an FDR < 0.1 between non-regressors and regressors, which reached statistical significance; cg13556949; *TULP2* (Fig. 3). The median *β* value for this site at baseline was 0.767 in progressors, and 0.779 in regressors. In the follow-up sample, the median *β* value for non-regressors increased to 0.860, while in comparison was steady in regressors, at 0.775 (Fig. 3). When a common threshold (p < 0.00001) was applied across *β* values and M values for the delta, two further CpG sites met this threshold (cg06616198, cg23054188) (Fig. 2C).

## Discussion

We have conducted the first EWAS in patients managed with active surveillance for CIN2 and identified novel CpG sites associated with non-regression (progressing or persistent CIN2), three of which remain statistically robust after correcting for multiple testing. *ALDH9A1* methylation at baseline was significantly increased in women whose lesions failed to regress within 24 months (FDR 0.068), and *MED25* methylation was significantly lower in samples from lesions imminently regressing (FDR 0.040). A larger *TULP2* methylation delta (Δ, change in methylation between baseline and follow-up samples at 12 months) was associated with non-regression (FDR 0.030).

Aldehyde dehydrogenases play a vital role in cell proliferation, differentiation and survival [49], thus biological plausibility exists for a biomarker involving methylation at this site. Additionally, they have recently been identified as potential biomarkers for solid tumours including cervical cancer [50]. Hypermethylation of the promoter region by HPV and lower expression of ALDH1A2 was associated with worse disease-specific survival in 297 invasive cervical cancers [51], and *ALDH1L1* was frequently methylated or deleted in 48 cervical cancer samples when compared to paired normal samples [52]. Examining *ALDH9A1* on the Human Protein Atlas (HPA) reveals a worse 5-year survival in 291 cervical cancers with a low expression (60% vs 71%, p = 0.032) [53]. Cg1275493 is situated in Open Sea on chromosome 1, with its closest associated gene being *ALDH9A1*; speculatively, increased methylation here may be leading to reduced gene expression in our cohort. This may implicate aldehyde dehydrogenases as important components in the cell’s mechanism to reverse the beginnings of carcinogenesis, which is in line with information on aldehyde dehydrogenases currently available in the literature [50, 51].

*MED25* methylation was lower in samples regressing imminently, i.e. within the following 12 months. MED25 has been identified as a transcription factor, suggested to play a crucial role in chromatin remodeling [54, 55]. Frameshift mutations of *MED25* have been found in colorectal cancers [56]. The HPA cohort 5-year survival rate for cervical cancer is 75% with high expression of MED25, compared to 58% with a low expression (p = 0.0052) [57]. MED25 has also been shown to interact with viral proteins including Herpes Simplex virus activator [58], Respiratory Syncytial Virus NS1 Protein [59], and Varicella Zoster virus [60]. Viral proteins can target the mediator complex to control both viral and host gene expression [61]. The possible role of MED25 in viral-human-interaction and cancer development is corroborated by its significantly worse survival in the HPA database in a population of 499 people with head and neck cancers (5-year survival 59% for high expression versus 41% for low expression, p < 0.0001) [62]. A lower level of methylation at this site in samples that went on to regress imminently may suggest that the mechanistic function of MED25 facilitates reversal of virally-induced histological change. If future work corroborated the role of MED25 as a vital protein in human-viral interactions, this could be a possible pathway on which to target anti-viral drug development.

Methylation levels at *TULP2* increased in non-regressors between baseline and follow-up, remaining stable in those that regressed. *TULP2* encodes proteins of an unknown function, and has its highest expression in testis tissue [63], and testicular cancer [64], as well as lymphoma and other cancer cell lines [65]. TULP2 expression is enriched in plasmacytoid dendritic cells [66], which have been shown to play a role in the immune response to HPV [67]; speculatively, the increase in methylation may represent a decreasing relative percentage of specific immune cells, as the immune system fails to prevent viral carcinogenic transformation within the cervix. Novel future work using flow cytometry in serial samples of women with CIN could help provide more clarity on the directional impact of various immune cell types on disease progression.

Previous work on DNA methylation in cervical cancer has primarily focused on diagnostics [68], although in recent years there are increasing studies examining DNA methylation as a prognostic tool, including in CIN. Louvanto et al. [29] applied the S5 classifier to women managed with active surveillance for CIN2, and found that when combined with high-grade cytology, the Area Under the Curve (AUC) for identifying progression as compared to regression was good, 0.735 (95%CI 0.621- 08.49). However, the clinically-relevant cut-off for CIN2 would be non-regression; as persistent CIN2 after 24 months warrants excision [22].The classifier’s ability to identify non-regressors reduced significantly, reaching a maximum AUC of 0.666 (95%CI 0.580-0.752) when combined with other features. Kremer et al. applied the Qiasure Methylation test by Qiagen (Germany) to 114 women aged 20-53 with CIN2 or CIN3 [44]. This commercially-developed methylation test based on two genes (*FAM19A4/miR124-2)* was taken at baseline; more than half of methylation-positive clinician-samples regressed; 51.4% (95%CI 34.6-65.9). Similarly to the S5 classifier [69], the Qiasure test was developed to diagnose high-grade CIN [70], rather than to predict disease progression. Likewise, Hoyer *at al* [71] examined the use of commercially-developed diagnostic methylation test of six genes (*ASTN1*, *DLX1*, *ITGA4*, *RXFP3*, *SOX17* and *ZNF671*), known as GynTect, in 24 patients with CIN2 who underwent active surveillance for up to 24 months. They found that 67% (12/18) GynTect-negative CIN2 patients regressed, and 75% (4/6) GynTect-positive CIN2 patients regressed, and therefore this marker appears unlikely to be clinically useful in predicting non-regression. Logically, it seems unlikely that biomarkers aimed at diagnosing CIN2, which would aim to be positive in the majority of CIN2 lesions, would accurately predict non-regression in this cohort.

The first EWAS in longitudinal cervical samples was undertaken by Bukowski et al. in 289 patients with normal or CIN1 histology at baseline, 15 of whom went on to progress to CIN2 + ^47^. This study was underpowered; splitting their cohort into a training and a testing set (with only 7 progressors in the training set and 8 in the testing set), 22 CpGs which met FDR significance in the training set produced a regression co-efficient of -0.04 in the test set. However, given the cost-effectiveness^70 71^ and high sensitivity of hrHPV^72-74^ testing to detect CIN2 + , the clinical application of identifying the few patients with normal/CIN1 histology who may develop CIN2 prior to the next screening round appears limited.

A strength of our study is that we have performed one of the most validated EWAS arrays, Illumina MethylationEPIC BeadChip 850k Methylation, aiming to identify novel and likely unstudied epigenome markers, which facilitated discovery of the CpG sites most specific to the clinical question. More extensive methylation data could be obtained using whole genome direct sequencing techniques, although the extra CpGs assessed using this method are less well-studied than the Illumina technique, and are less likely to provide meaningful information. Our study is limited by the small number included, particularly of non-regressors (n = 12), and with this, low statistical power. Using samples from this unique cohort of women with CIN2 who underwent active surveillance and have a known outcome that translates well into clinical practice makes our study valuable, although the rarity of this kind of cohort has precluded replication. A possible further limitation is the nature of the included cohort being young, although active surveillance is more likely to be practiced in younger women, and there is no evidence to suggest that these particular identified methylation sites would change dramatically between the age of 20 and 30. Age-related epigenetic clocks do exist, and these particular sites were not identified in one of the most well-established clocks [72, 73]. Use of a 12 month threshold to subdivide regressors is arbitrary, given that most guidelines for CIN2 permit 24 months of active surveillance, however, in a recently published large cohort study, 90% of women who regressed did so within the first 12 months [15]. Additionally, the ability to predict regression within 12 months may be of value to women who do not wish to undergo 24 months of intensive follow-up. The pathological diagnostic process used in the original study is representative of current practice globally; although the recent LAST guidelines now describe the use of p16 to aid in diagnosis, availability remains limited and the recommendation is only for equivocal cases [74]. Finally, the majority of currently established diagnostic methylation tests comprise a number of CpGs, and it is likely that a similar principle would also need to be applied to develop a robust prognostic test in this setting.

Over half of CIN2 lesions may regress spontaneously [14, 15], therefore the decision to treat surgically or to actively monitor must take into account the patient’s age, clinical features including size of the lesion, and future fertility wishes [75–78]. Factors such as age, HPV type, and cytology should be considered, although evidence for strong associations appears conflicting in current literature [22, 23, 79, 80]. One study in Denmark found that HPV16 was associated with non-regressive lesions [23], although another study looking at Finnish women did not find significance of HPV type at baseline [80]. As genotyping is not practiced in all settings including the UK, we aimed to explore an alternative molecular marker, of which methylation may be a good avenue to pursue. For patients who wish to undergo active surveillance, which may reduce the chance of obstetric complications in future pregnancies [18, 19, 81], a biomarker that could accurately predict non-regression could provide the necessary reassurance to continue with active surveillance, or sufficient evidence to recommend surgical excision. An accurate biomarker is urgently needed to guide decision-making for the 2-3% of women attending cervical screening programmes diagnosed with CIN2 per year [82, 83], and this work highlights the potential of novel DNA methylation markers such as *ALDH9A1* in answering this specific clinical question. It is worth noting that the change in methylation, although statistically significant, is small, and the translation of these methylated sites into binary biomarkers could be challenging. Methylation at *ALDH9A1* appears more translatable than *MED25*, given the lack of overlap in IQR (Fig. 1A and B). It is possibly more likely that a scaled methylation biomarker would be taken into consideration in the context of the whole clinical picture, including the clinical features used currently, such as size of the lesion, and the age of the woman. For those eligible for active surveillance, higher methylation could point to a lower chance of success, and aid clinical decision making.

In our cohort of 58 young women who underwent active surveillance for CIN2, methylation at novel CpG sites *ALDH9A1* and *MED25* were associated with non-regression at 24 months and non- regression within the subsequent 12 months respectively. Methylation levels at *TULP2* increased significantly between the baseline and follow-up samples in patients whose CIN2 lesions did not regress, in comparison to those that regressed. Molecular markers accurately predicting non- regression could facilitate treatment stratification for women with CIN2. This could allow those who have a high chance of regression to avoid surgical treatment of the cervix and its sequelae, whilst ensuring those less likely to regress can be treated in a timely manner, avoiding the need for multiple visits and the risk of disease progression. Further work using similar epigenome-wide techniques is needed to validate these findings in larger prospective cohorts. Additionally, mechanistic studies that obtain parallel methylation and expression data may help to deepen understanding of pro- and anti-carcinogenic pathways.

## Supplementary information

Supplementary Figure Legends

Figure S8

Figure S7

Figure S6

Figure S5

Figure S3

Figure S2

Figure S1

Figure S4

## Data Availability

Raw epigenome wide data available on request.

## Code availability

Code available on request.
