# Supplementary material for: Methylation biomarkers in non-regressive cervical intraepithelial neoplasia grade 2 lesions: an epigenome wide association study
Source: Br J Cancer. 2026 Apr 11;135(1):118–26. doi: 10.1038/s41416-026-03391-4 (PMC13269546; doi:10.1038/s41416-026-03391-4)
Supplement: Supplementary file 1 — Supplementary Figure Legends [file 41416_2026_3391_MOESM1_ESM.docx]

**Supplementary Figure Legends**

Figure S1: Overall distribution of Beta values, stratified by sample status (non-regressors = red, late regressor = orange, immediate regressor = green).

Figure S2: Overall distribution of Beta values, stratified by timepoint (baseline = red,12 month follow-up = blue).

Figure S3: A SNP Heatmap to confirm the identity of the follow-up sample

Figure S4 and S5: Principle components analysis. Figure S4: PC3 most significant largely consisting of chip and plate. Figure S5: once chip and plate have been adjusted for, PC4, PC8 and PC9 can be seen as significant, which are not associated with sample status

Figure S6: Q-Q plot: Non-regressors vs all Regressors (<24 months)

Figure S7: Q-Q plot: Non-regressors vs all Imminent Regressors (<12 months)

Figure S8: Q-Q plot: Delta (change in methylation) - Non-regressors vs regressors
