# Supplementary figures and images for: Methylation biomarkers in non-regressive cervical intraepithelial neoplasia grade 2 lesions: an epigenome wide association study
Source: Br J Cancer. 2026 Apr 11;135(1):118–26. doi: 10.1038/s41416-026-03391-4 (PMC13269546; doi:10.1038/s41416-026-03391-4)

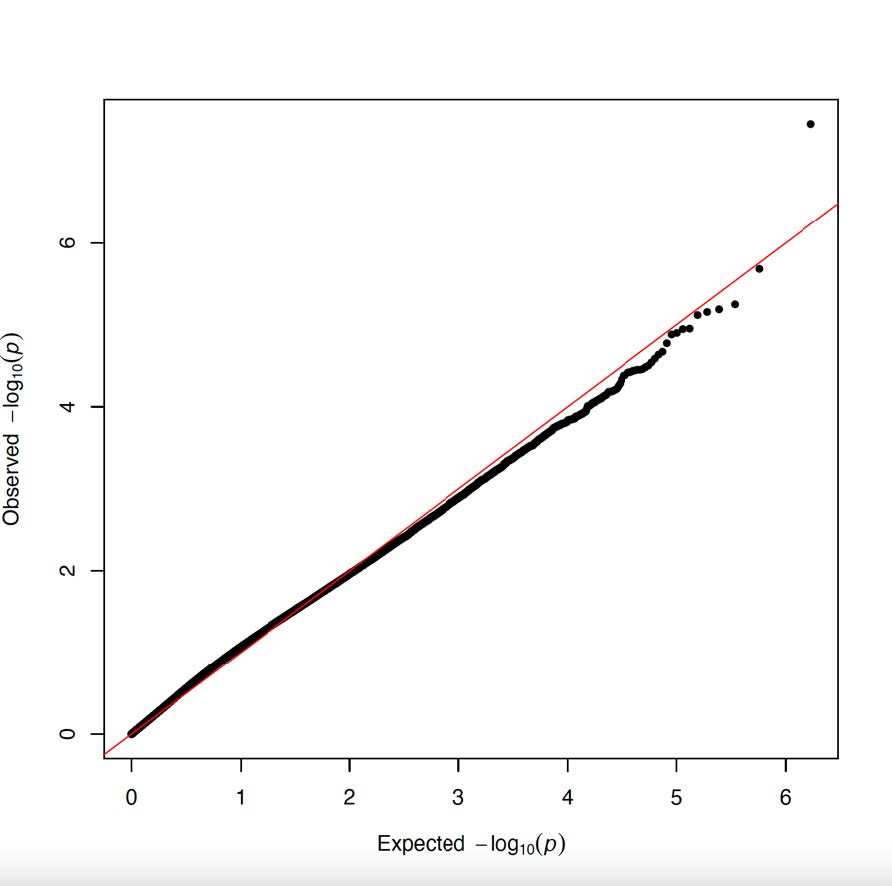

Supplement: Supplementary file 2 — Figure S8 [file 41416_2026_3391_MOESM2_ESM.jpg]

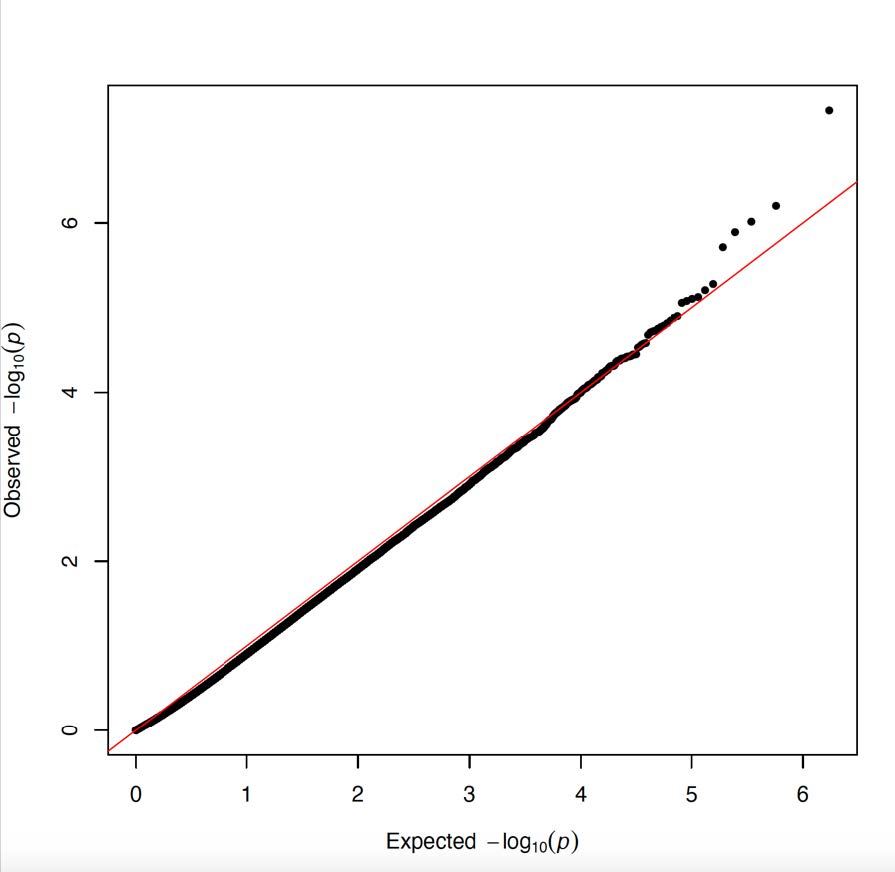

Supplement: Supplementary file 3 — Figure S7 [file 41416_2026_3391_MOESM3_ESM.jpg]

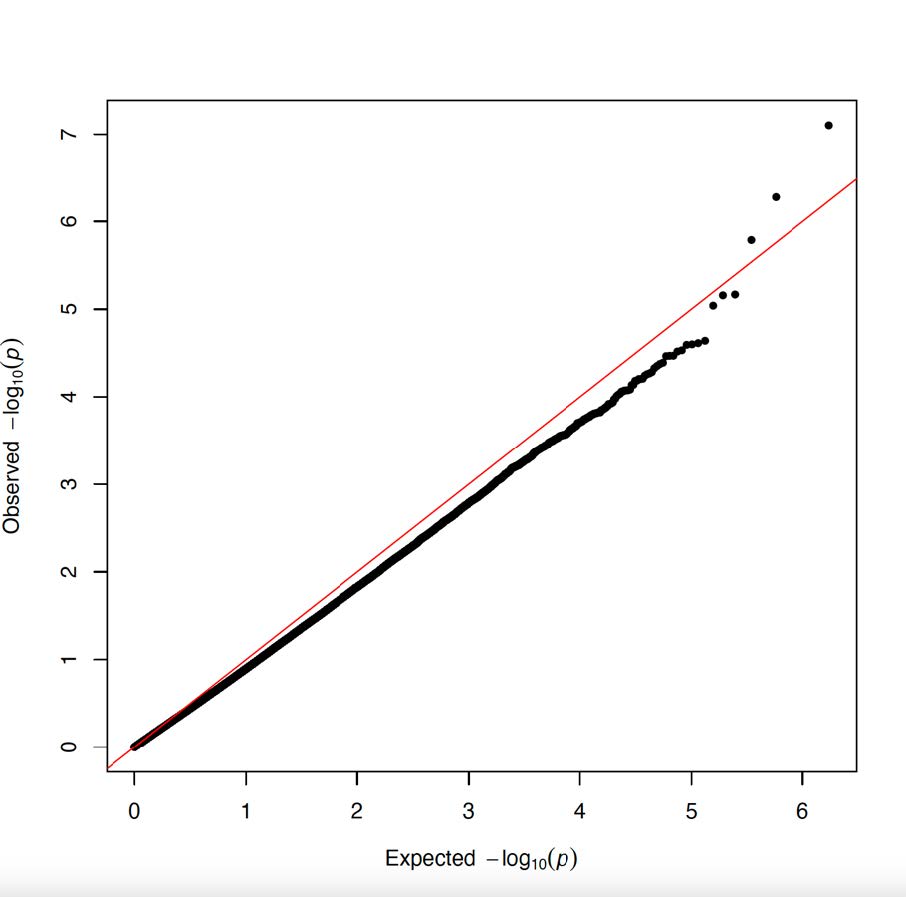

Supplement: Supplementary file 4 — Figure S6 [file 41416_2026_3391_MOESM4_ESM.jpg]

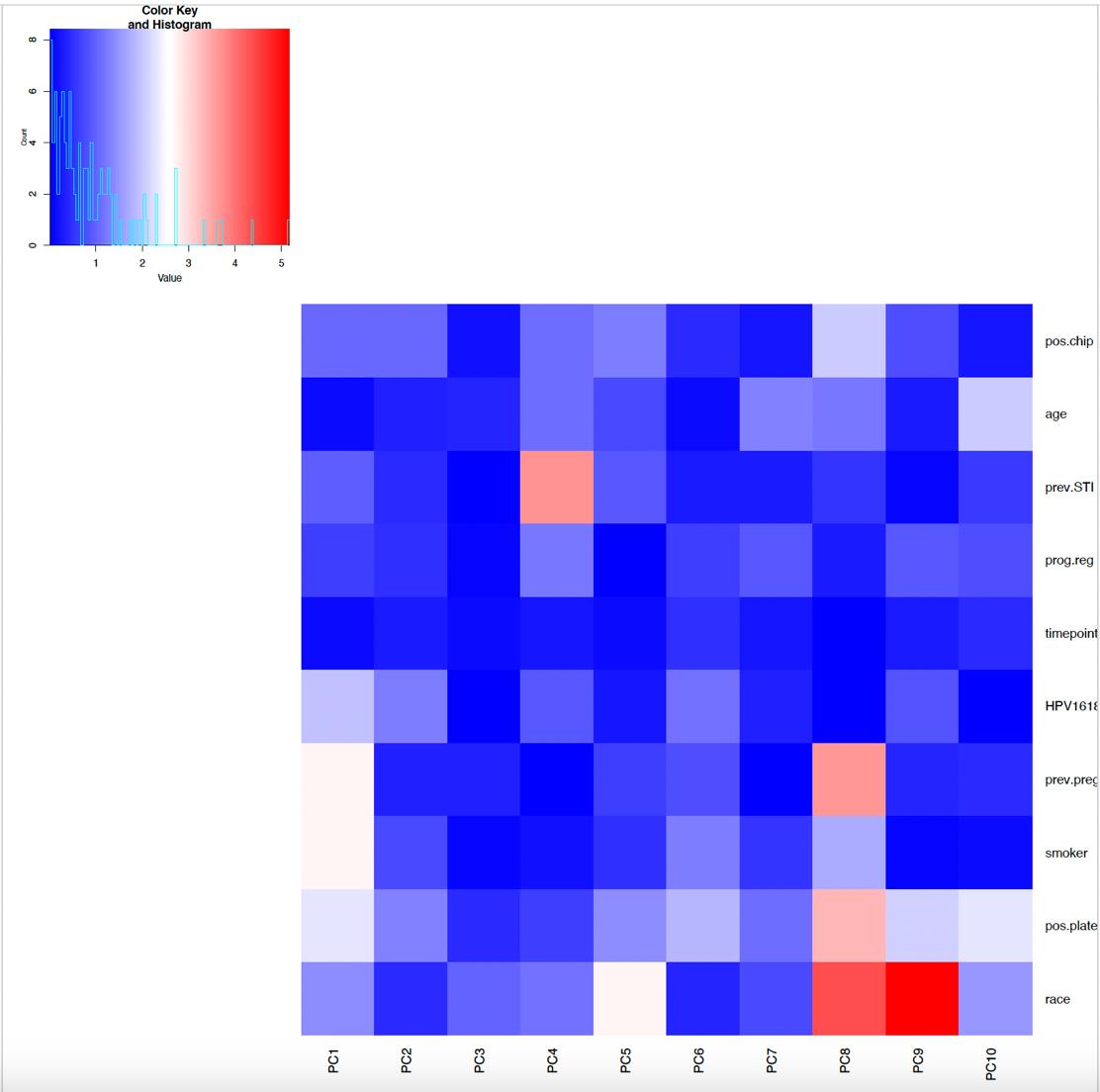

Supplement: Supplementary file 5 — Figure S5 [file 41416_2026_3391_MOESM5_ESM.jpg]

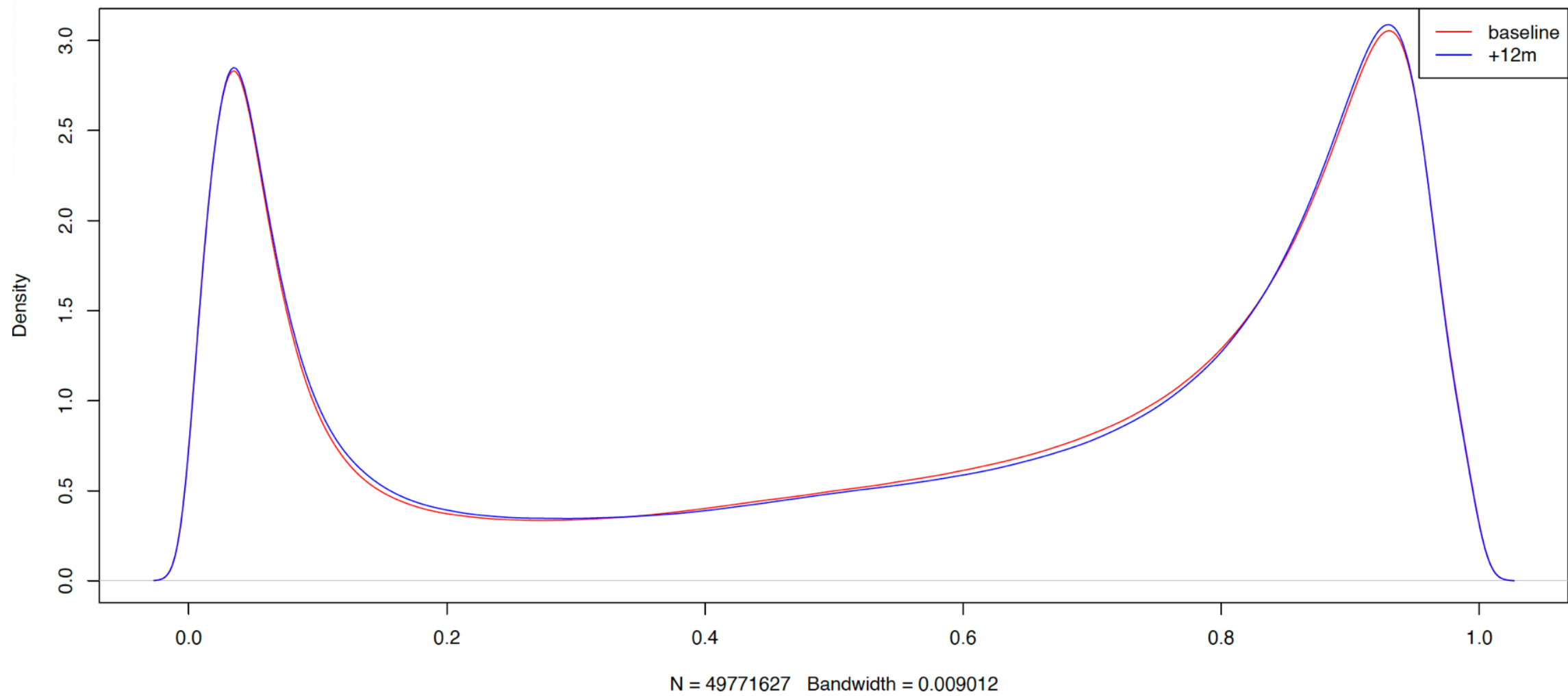

Supplement: Supplementary file 7 — Figure S2 [file 41416_2026_3391_MOESM7_ESM.pdf]

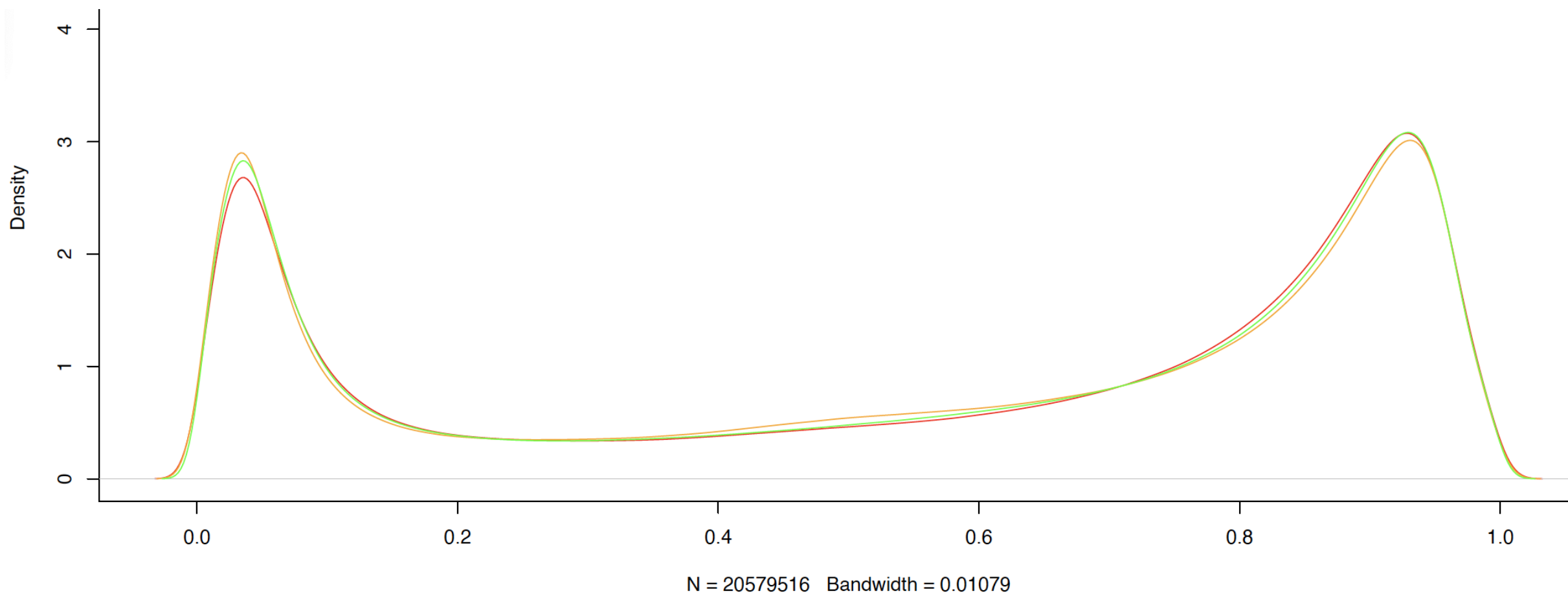

Supplement: Supplementary file 8 — Figure S1 [file 41416_2026_3391_MOESM8_ESM.pdf]

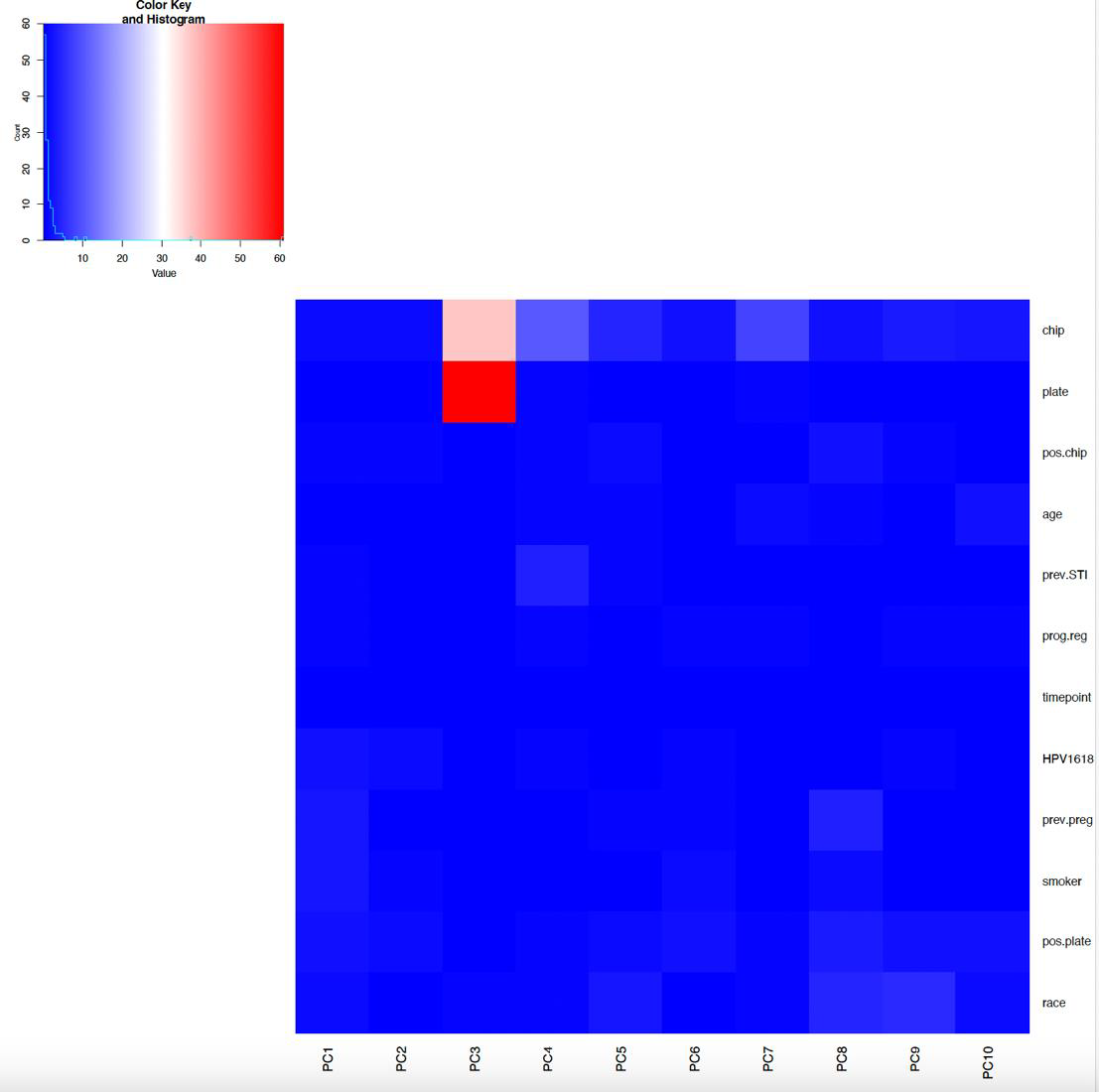

Supplement: Supplementary file 9 — Figure S4 [file 41416_2026_3391_MOESM9_ESM.png]
