# Supplementary material for: Methylation biomarkers in non-regressive cervical intraepithelial neoplasia grade 2 lesions: an epigenome wide association study
Source: Br J Cancer. 2026 Apr 11;135(1):118–26. doi: 10.1038/s41416-026-03391-4 (PMC13269546; doi:10.1038/s41416-026-03391-4)

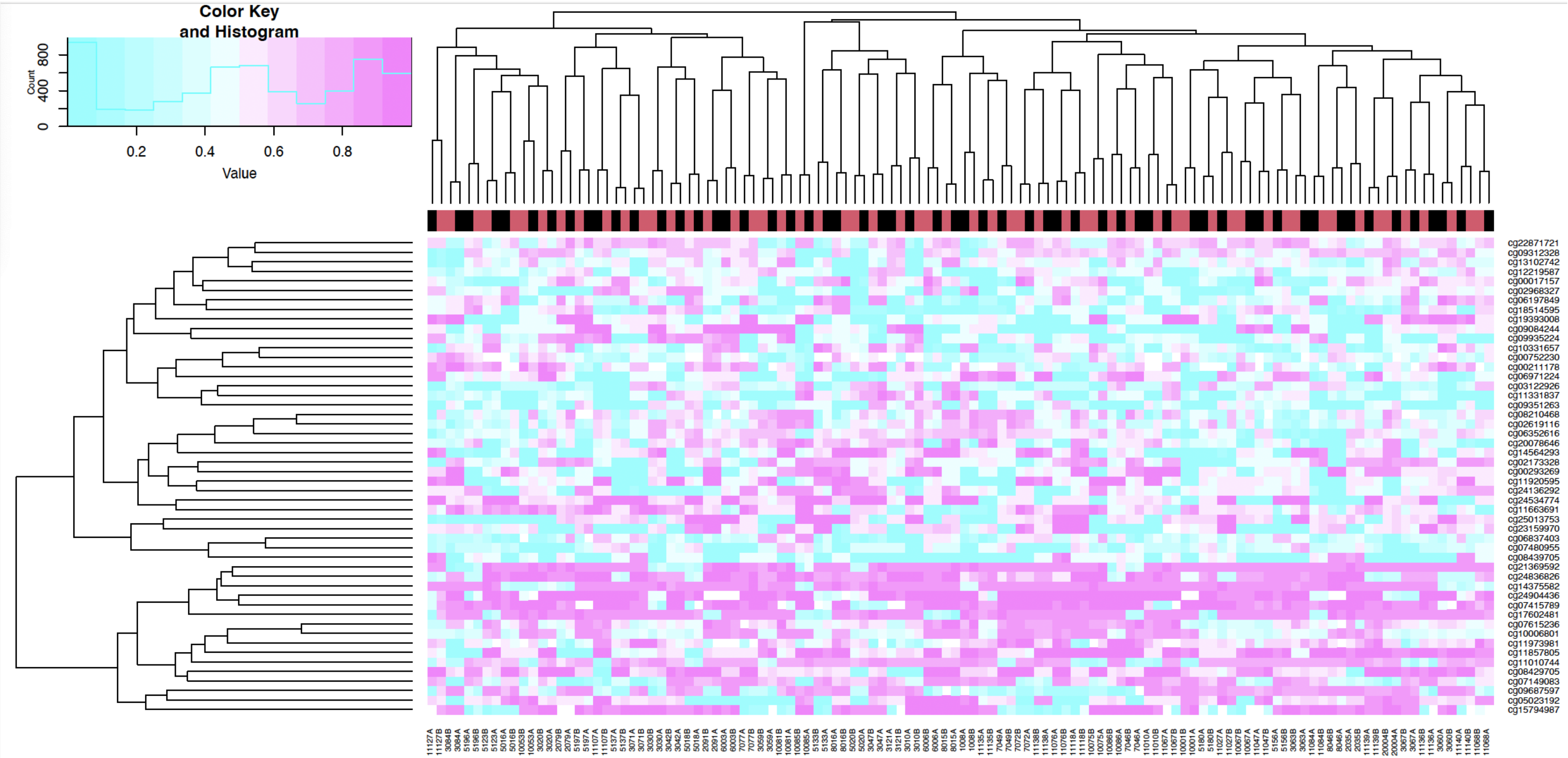
Color Key
and Histogram
Count
0
400
800
0.2
0.4
0.6
0.8
Value
cg22871721
cg09312328
cg13102742
cg12219587
cg00017157
cg02968327
cg06197849
cg18514595
cg19393008
cg09084244
cg09935224
cg10331657
cg00752230
cg00211178
cg06971224
cg03122926
cg11331837
cg09351263
cg08210468
cg02619116
cg06352616
cg20078646
cg14564293
cg02173328
cg00293269
cg11920595
cg24136292
cg24534774
cg11663691
cg25013753
cg23159970
cg06837403
cg07480955
cg08439705
cg21369592
cg24836826
cg14375582
cg24904436
cg07415789
cg17602481
cg07615236
cg10006801
cg11973981
cg11857805
cg11010744
cg08429705
cg07149083
cg09687597
cg05023192
cg15794987

Supplementary Figure 3 legend: *Principle components analysis*. *Figure 3A: PC3 most significant largely consisting of chip and plate. Figure 3B: once chip and plate have been adjusted for, PC4, PC8 and PC9 can be seen as significant, which are not associated with sample status.*

Figures S4 and S5: Principle Components Analysis

S4

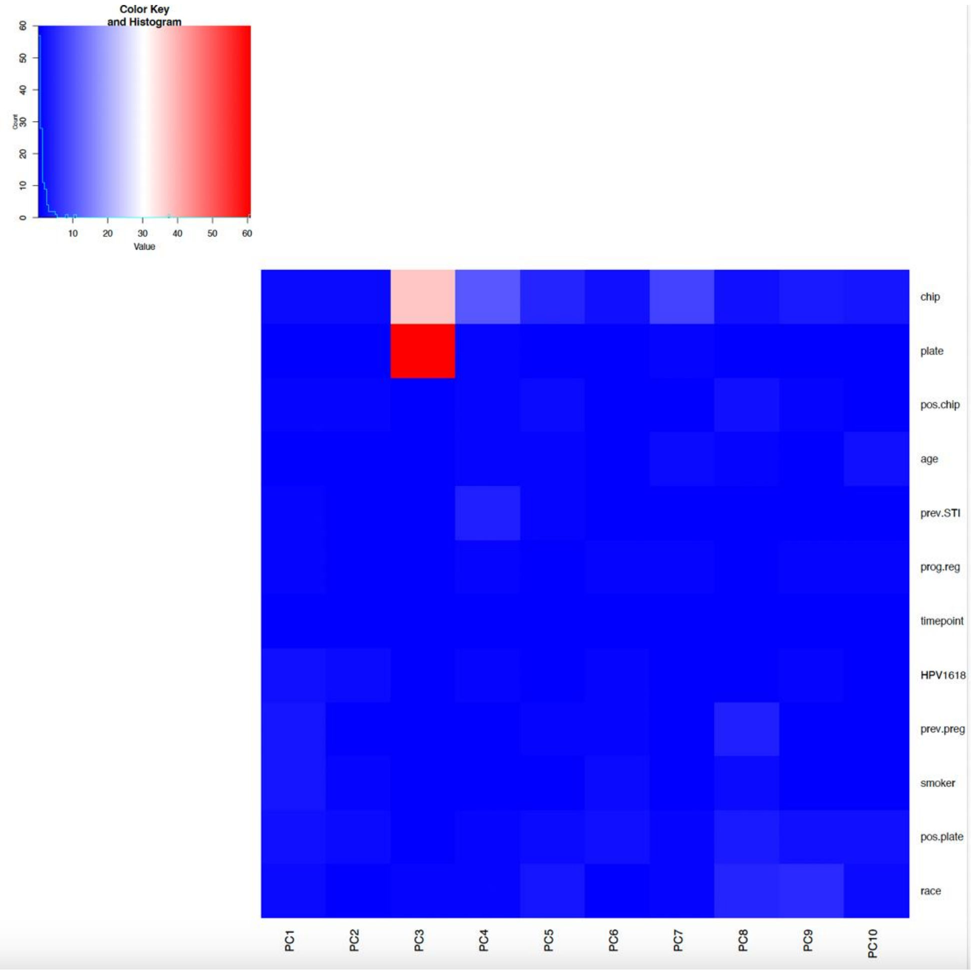

S5

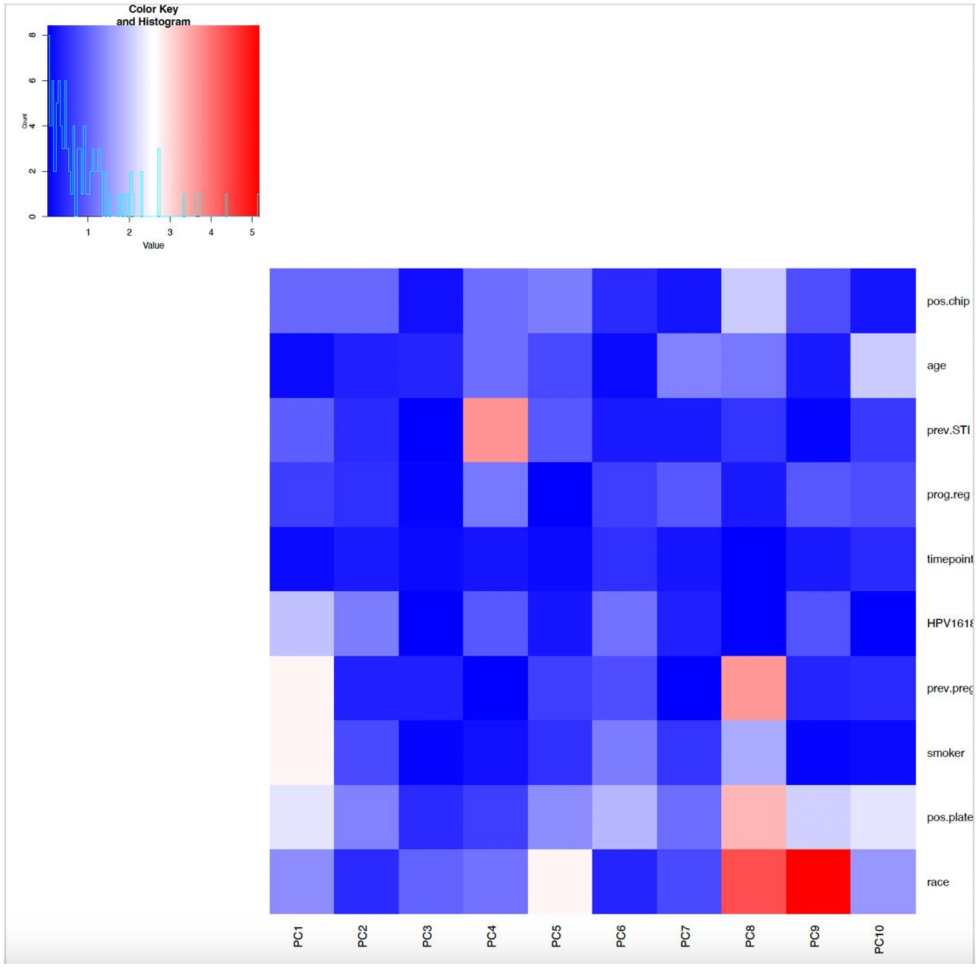

Figure S6-8: *Q-Q plots created for each statistical analysis.*

S6

Non-regressors vs all Regressors
(<24 months)

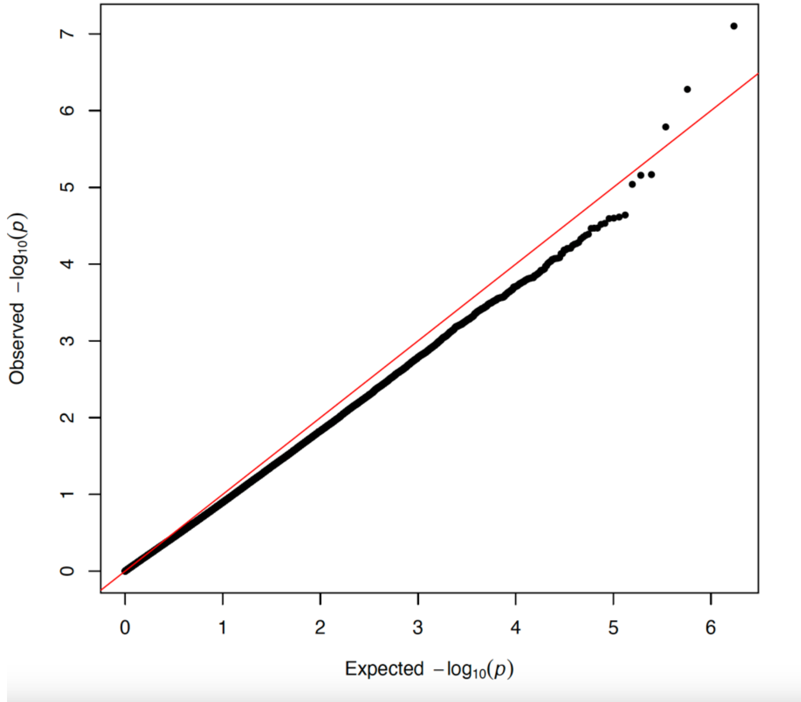

S7

Non-regressors vs regressors
(<12 months)

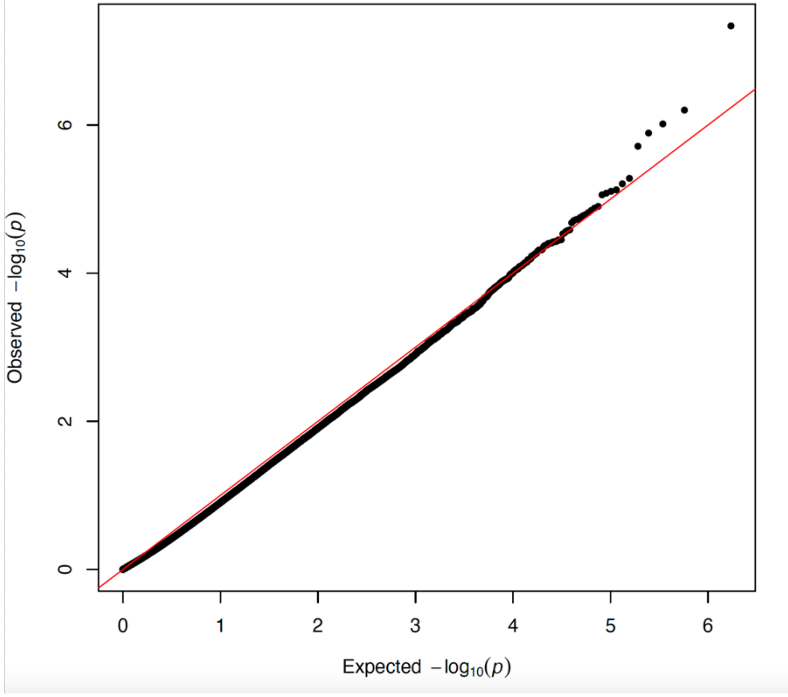

S8

Delta (change in methylation):
Non-regressors vs regressors

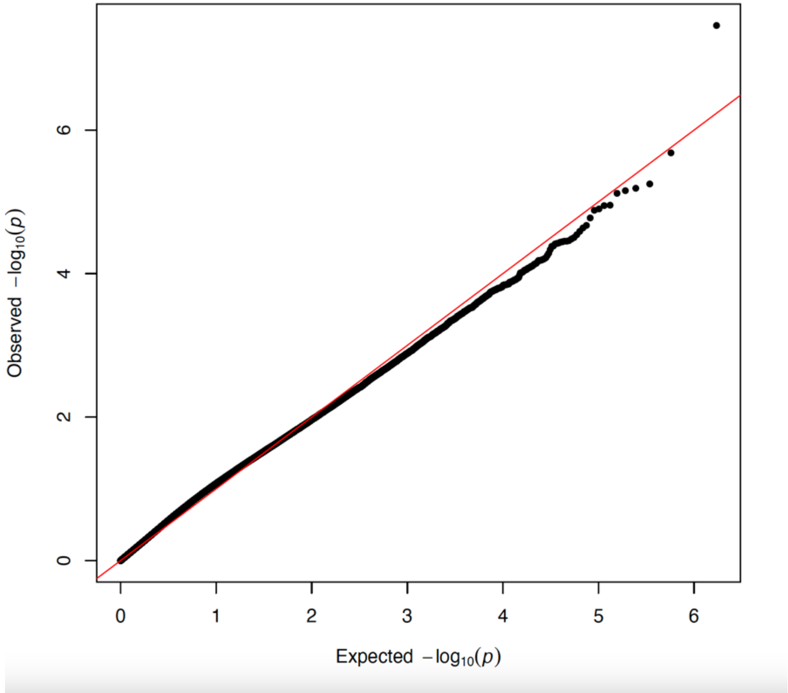

Supplement: Supplementary file 6 — Figure S3 [file 41416_2026_3391_MOESM6_ESM.pdf]
